# Interaction Kinetics of Individual mRNA-Containing Lipid Nanoparticles with an Endosomal Membrane Mimic: Dependence on pH, Protein Corona Formation, and Lipoprotein Depletion

**DOI:** 10.1021/acsnano.2c04829

**Published:** 2022-12-13

**Authors:** Nima Aliakbarinodehi, Audrey Gallud, Mokhtar Mapar, Emelie Wesén, Sahar Heydari, Yujia Jing, Gustav Emilsson, Kai Liu, Alan Sabirsh, Vladimir P. Zhdanov, Lennart Lindfors, Elin K. Esbjörner, Fredrik Höök

**Affiliations:** † Division of Nano and Biophysics, Department of Physics, Chalmers University of Technology 41296 Göteborg, Sweden; ‡ Division of Chemical Biology, Department of Biology and Biological Engineering, Chalmers University of Technology, 41296 Göteborg, Sweden; § Advanced Drug Delivery, Pharmaceutical Sciences, R&D, AstraZeneca, 43181 Gothenburg, Sweden; ∥ Boreskov Institute of Catalysis, Russian Academy of Sciences, Novosibirsk 630090, Russia

**Keywords:** ionizable lipid nanoparticle, mRNA delivery, endosomal membrane, protein corona, lipoprotein

## Abstract

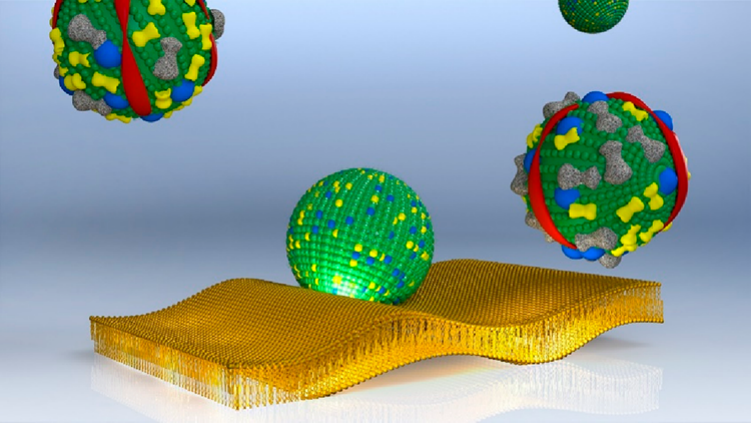

Lipid nanoparticles (LNPs) have emerged as potent carriers for mRNA delivery, but several challenges remain before this approach can offer broad clinical translation of mRNA therapeutics. To improve their efficacy, a better understanding is required regarding how LNPs are trapped and processed at the anionic endosomal membrane prior to mRNA release. We used surface-sensitive fluorescence microscopy with single LNP resolution to investigate the pH dependency of the binding kinetics of ionizable lipid-containing LNPs to a supported endosomal model membrane. A sharp increase of LNP binding was observed when the pH was lowered from 6 to 5, accompanied by stepwise large-scale LNP disintegration. For LNPs preincubated in serum, protein corona formation shifted the onset of LNP binding and subsequent disintegration to lower pH, an effect that was less pronounced for lipoprotein-depleted serum. The LNP binding to the endosomal membrane mimic was observed to eventually become severely limited by suppression of the driving force for the formation of multivalent bonds during LNP attachment or, more specifically, by charge neutralization of anionic lipids in the model membrane due to their association with cationic lipids from earlier attached LNPs upon their disintegration. Cell uptake experiments demonstrated marginal differences in LNP uptake in untreated and lipoprotein-depleted serum, whereas lipoprotein-depleted serum increased mRNA-controlled protein (eGFP) production substantially. This complies with model membrane data and suggests that protein corona formation on the surface of the LNPs influences the nature of the interaction between LNPs and endosomal membranes.

Recent advances in the design of oligonucleotide-containing lipid nanoparticles (LNPs) enabling protein production in vivo have contributed to the emergence of the clinically approved and now widely used mRNA-based COVID-19 vaccines and are of high relevance in the context of pharmaceutical interventions for multiple diseases where therapeutic alternatives are sparse.^1,2^ LNP-mediated oligonucleotide delivery is critically dependent not only on successful cellular uptake but also on the subsequent cellular processes. One of the key steps in delivery is associated with entrapment of LNPs at anionic membranes of endosomes^3^ accompanied by membrane destabilization to induce mRNA escape.^4^ Still, utilizing the most prominent LNPs for delivery, endosomal mRNA escape occurs at a low efficiency, a few percent at best,^5,6^ and has been identified as the key rate-limiting step.^5−8^ Accordingly, understanding the mechanistic nature of endosomal escape is of great interest.

However, investigations of this type are complicated by the fact that prior to cellular uptake, LNPs become spontaneously coated with proteins from biological fluids in a complex and far from understood manner.^9,10^ This so-called protein corona on the surface of LNPs is in fact often a requirement for efficient cellular uptake,^11^ and lipoproteins, in particular apolipoprotein E (ApoE), have been identified as particularly crucial, likely by mediating binding to LDL receptors residing in plasma membranes.^12,13^ Significantly less is known, however, regarding the possible impact of the protein corona on endosomal processing of LNPs, and how endosomal acidification and enzymatic degradation of the protein corona may influence the critical escape events. We have recently shown that LNP-mediated mRNA uptake and subsequent protein translation depend on the duration of LNP-serum preincubation, and hence protein corona maturation, prior to cellular exposure,^14^ which suggests that the protein corona formation deserves special attention.

Conceptual mechanisms of endosomal escape are typically identified using advanced cellular assays.^4,15,16^ Detailed insights regarding the biomolecular mechanisms controlling the interaction between biological nanoparticles and cellular membranes have also been gained by complementing live cell data with various cell membrane mimics using a broad arsenal of quantitative bioanalytical tools,^17−20^ some of which offer single nanoparticle resolution, such as atomic force microscopy^21,22^ and total internal reflection fluorescence (TIRF) microscopy.^23,24^ Most of the latter studies have been focused on the interaction between biological nanoparticles, such as viruses and LNPs, and mimics of the outer plasma membrane, to gain information on specific ligand–receptor interactions,^25−27^ including multivalent interactions with cell membrane receptors^28−30^ and how this type of interaction can be promoted or inhibited.^31,32^ With exception for TIRF-assisted investigations of pH induced fusion between viruses and lipid vesicles mimicking the endosomal membrane,^33^ significantly less efforts have been put into the design of endosomal membrane mimics and investigations of how the interaction between nanoparticle-based oligonucleotide carriers and the endosomal membrane varies during the unidirectional maturation of endosomes, a process that is accompanied by a gradual increase in the negative charge of their membranes^34^ and a reduction of the luminal pH from ∼7 to <5.^35^ To this end, Tamaddon et al.^36^ investigated the release mechanism of oligodeoxynucleotides from permanently charged cationic liposomes upon binding to vesicles designed to mimic the anionic properties of the endosomal membrane, while Peetla et al.^37^ investigated the association of cationic liposomes with an endosomal model membrane to clarify the biomechanics and thermodynamics of endocytosis and endosomal escape. Recently, Spadea et al. investigated LNP interaction with an anionic lipid monolayer formed at an air–water interface,^20^ revealing pH-dependent surface-pressure and density changes consistent with lipid transfer between adsorbed LNPs and the anionic lipid film.

Inspired by the latter investigations and the success of the LNP design principle^38^ behind, for example, Onpattro, the first FDA-approved nucleic acid-based therapy to cure hereditary transthyretin amyloidosis,^39^ and the COVID-19 vaccines Comirnaty and Spikevax (mRNA-1273) from Pfizer/BioNTech^40^ and Moderna,^41^ respectively, our work focuses on PEGylated LNPs containing ionizable lipids. Specifically, we utilized high resolution fluorescence microscopy to investigate the pH dependence of LNP binding to an anionic model membrane using an LNP formulation containing the amine-modified ionizable lipid DLin-MC3-DMA (MC3),^2,42,43^ which is neutral at pH 7 but become positively charged at lower pH via protonation of the amine moieties.^2^ Upon endocytosis, entrapment of LNPs at anionic membranes of endosomes^3^ is believed to be mediated by ion pair formation between protonated MC3 and the anionic endogenous endosomal phospholipids eventually resulting in a configuration that promotes release of mRNA into the cytoplasm of the target cells,^44,45^ a process that is thought to be facilitated by the cone-shaped structure of the MC3 promoting an inverted nonbilayer configuration that destabilizes the endosomal membrane.^45^

To resolve LNP binding to a negatively charged supported lipid bilayer (ncSLB), formed on the glass floor of the fluidic channel, in real time and with single LNP resolution, we designed a microfluidic-based assay combined with TIRF imaging. Emphasis was put on the nature of the pH-dependent LNP interaction with the ncSLB, and how it is influenced by preincubation of the LNPs with (i) untreated, (ii) lipoprotein-depleted, and (iii) lipoprotein-saturated serum. Together with complementary live cell experiments to measure LNP uptake and mRNA delivery (here eGFP production), the work provides insights into the mechanistic aspects of LNP attachment to and disintegration on the ncSLB as well as the role of protein corona formation, in general, and particularly the role of lipoproteins on these crucial events.

## Results and Discussion

**Figure 1:**
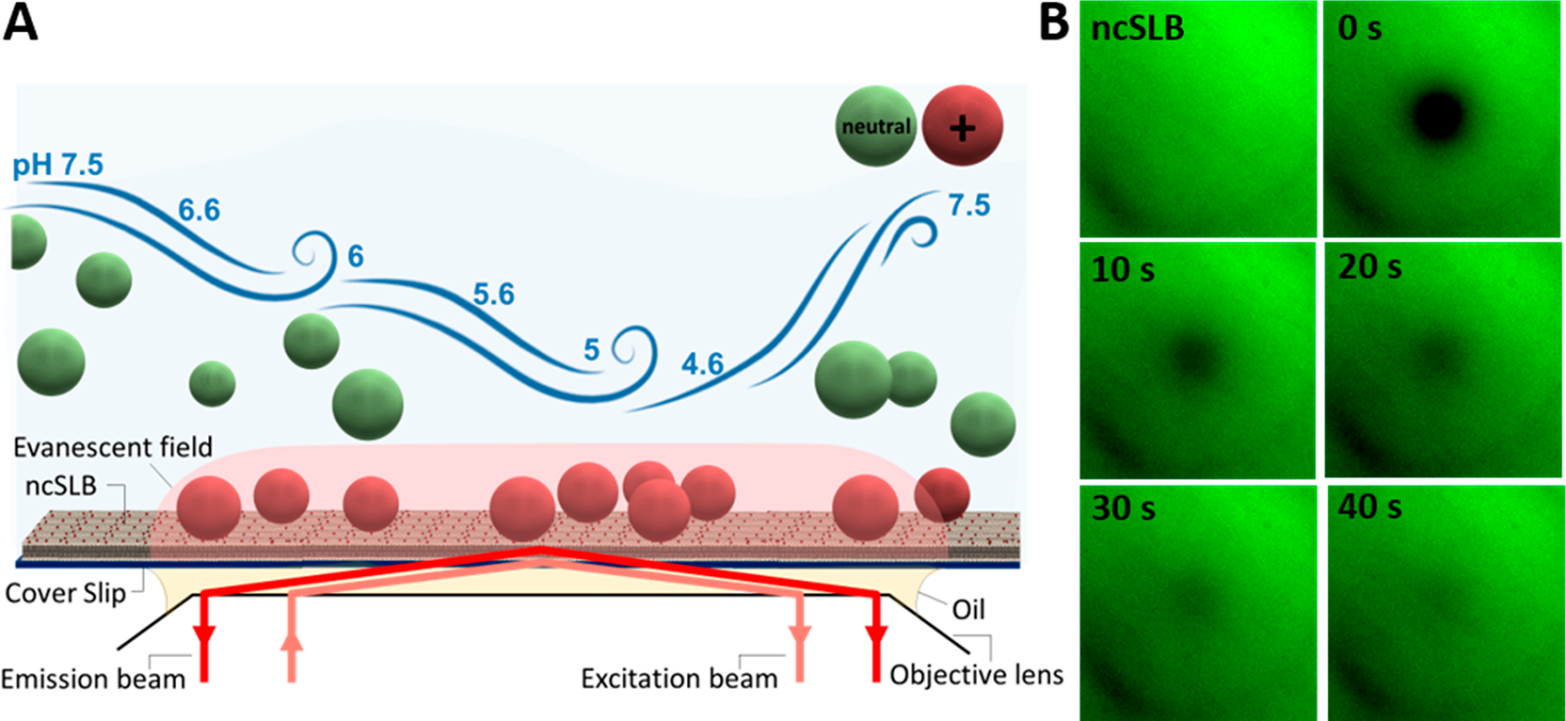
(A) Schematic representation of the endosomal model system consisting of a ncSLB formed on the coverslip inside a microfluidic channel and coupled to a TIRF microscope for time-resolved surface-sensitive imaging. (B) TIRF image of a ncSLB prior to and after photo bleaching in time steps of 10 s from the FRAP analysis at pH 7.5, where *t* = 0 corresponds to the time point at which the bleaching was finished. Image sizes are 80 × 80 μm^2^.

We developed an assay designed for temporal monitoring of single LNP binding events to a SLB designed to mimic the endosomal membrane. In this strategy, the ncSLB was formulated with POPC as the major lipid component and 6 mol % negatively charged POPS lipids to represent the anionic character of early to late endosomal membrane conditions.^34,46^ LNP binding was continuously monitored under gentle flow conditions at a constant LNP concentration using time-lapse TIRF microscopy of Cy5 labeled mRNA (the LNP cargo) as the pH was reduced in steps from 7.5 to 4.6, followed by a final rinsing step at pH 7.5, as schematically illustrated in Figure 1A. To characterize the ncSLB, a small fraction (0.5 mol %) of fluorescently labeled PE lipids was incorporated, and fluorescent recovery after photobleaching (FRAP) was performed as previously described.^47^ The analysis revealed a lipid diffusivity of 3.43 ± 0.5 μm^2^/s and an immobile fraction of 0.01 ± 0.02 (*n* = 3), with slightly increasing diffusivity and immobile fraction upon stepwise reduction in the pH (Supporting Information, Figure S1), which is consistent with formation of a continuous SLB across the entire pH range (Figure 1B).

To facilitate time-resolved visualization of the binding of individual LNPs (diameter ∼80 nm), 20% of the total mRNA cargo (∼16 mRNAs per LNP) was fluorescently labeled with Cy5, and the LNP concentration was adjusted to ∼0.7 × 10^9^ particles/mL. The LNP suspension was initially injected into a rectangular microfluidic channel (height 400 μm, width 3.8 mm) at pH 7.5 for 20 min at a flow rate of 150 μL/min using a syringe pump, chosen to provide laminar flow but still sufficiently fast liquid exchange over the sensing area (on the order of seconds) without inducing appreciable shear force (<1 fN) on the adsorbed LNPs (see Supporting Information, section 10 for estimate). This step was followed by consecutive LNP injections at continuous flow (150 μL/min) for 20 min at each consecutively decreased pH: 6.6, 6.0, 5.6, 5.0, and 4.6. Note that these LNPs had not been exposed to any serum proteins and that the PEGylated state is thus not representative of what would occur in a biological context, in which case the LNPs are coated with a protein corona, as analyzed further below.

**Figure 2:**
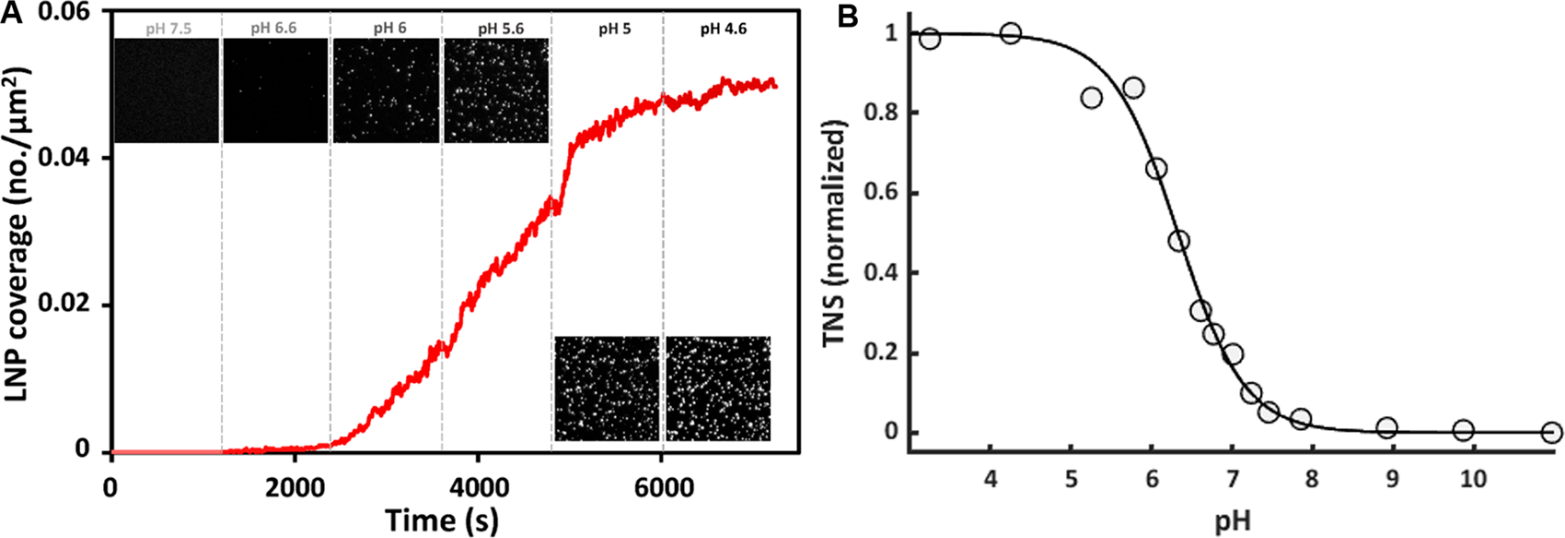
(A) Time-resolved binding of LNPs to a negatively charged SLB (ncSLB) upon gradual acidification of the buffer solution. The insets show TIRF micrographs (80 × 80 μm^2^) at the respective pH values just prior to the subsequent LNP injection. (B) In situ TNS fluorescence titration of pristine LNPs. Duplicate measurements were averaged and fitted to a three-parameter sigmoidal.

While there were virtually no LNP binding events at pH 7.5, a gradual increase in the rate of binding was observed as an increase in the number of Cy5-fluorescent objects at the SLB surface following acidification, which approached saturation at a coverage of ∼0.05 particles/μm^2^ at pH 5.0 (Figure 2A). In previous work an apparent isoelectric point of around 6 was estimated from zeta potential measurements for similar LNPs containing the same ionizable lipids, albeit at lower ionic strength (25 mM compared with 150 mM used in this study).^45^ While this coincides with the observed onset of LNP binding, both the zeta potential and the nature of electrostatic interactions depend on ionic strength. We therefore measured the relative surface charge of our LNPs at a representative ionic strength using the anionic fluorescent dye 2-(*p*-toluidino)-6-napthalene sulfonic acid (TNS), which undergoes a significant fluorescent enhancement when binding to positively charged lipids but that is nonfluorescent in solution.^48^ This approach has been previously used to characterize LNPs of the type used in this work.^49^ The TNS assay shows a relatively sharp transition around an inflection point at pH 6.35, with 20 and 80% ionization at around pH 7 and 5.5, respectively (Figure 2 B). In the LNP binding experiments, the electrostatic attraction continues to increase with decreasing pH down to pH 5.0, with an inflection point of the binding curve around pH 5.6 (Figure 2). This suggests that a significant amount of MC3 must be ionized before the electrostatic attraction becomes sufficiently large to overcome the steric repulsion expected between the shell of PEGylated lipids on the surface of the LNP and the ncSLB. Of note, LNPs preincubated in pH 4.6, which may induce structural alterations,^50,51^ displayed insignificant binding when exposed to the ncSLB at pH 7, demonstrating that the ionization is fully reversible and that the ncSLB is free from defects that induce nonspecific LNP binding (Supporting Movie S1).

It is also notable that there is a decrease in the rate of binding at pH 5.6 and 4.6. A plausible explanation of the reduction in the rate of LNP binding at pH 5.6 and 4.6 is that negatively charged lipids in the SLB become accumulated in the contact zones between the LNP and the SLB, while simultaneously, LNPs disintegrate and the corresponding positively charged MC3 (the MC3 structure together with the structure of the other lipid components in the LNP is shown in Figure S2) diffuse out from the contact zones, which would, in turn, balance negative charges in the ncSLB and suppress further attachment of LNPs.

To scrutinize these scenarios, it is worth noting that the saturated LNP coverage of ∼0.05 particles/μm^2^ is several orders of magnitude lower than the coverage of ∼100 particles/μm^2^ that corresponds to the jamming limit (∼54% surface coverage) of LNPs with a radius of 40 nm. To examine the magnitude of accumulation of negatively charged lipids in the LNP-SLB contact regions, we recall that with a fraction of POPS lipids η = 0.06 and an area per lipid *s* = 0.6 nm^2^, the average concentration of charged lipids in the SLB is *c* = η/*s* = 10^5^ μm^–2^. At an LNP coverage of ∼0.05 particles/μm^2^, this relatively high concentration of charged lipids in the SLB cannot be appreciably reduced if the LNPs remain nearly intact. If the LNPs instead undergo a significant collapse, but remain compact and confined near the attachment spot, the scale of the area of the LNP-SLB contact can be roughly estimated as *S* ≈ 4*R*^2^ = 6.4 × 10^4^ nm^2^, where *R* = 40 nm is the average LNP radius in solution. Lipids can to a first approximation be assumed to form a triangular lattice both in the SLB and the LNP. Due to steric repulsion, charged lipids are not expected to be located in the nearest-neighbor sites of such a lattice. This means that the minimal area per charged lipid should not be smaller than 3*s* = 1.8 nm^2^. Thus, the maximum number of charged lipids in the contact area can be estimated as *n*_∗_ = *S*/3*s* = 1.2 × 10^3^. Further, the average concentration of charged lipids (per μm^2^ of the SLB) associated with attached LNPs is consequently given by *c*_∗_ = *n*_∗_*C* = 60 μm^–2^, where *C* = 0.05 μm^–2^ is the LNP coverage at which the rate of LNP binding is suppressed. Under these conditions *c*_∗_ is around 3 orders of magnitude lower than *c* (the average concentration of charged lipids in the SLB) even for compactly collapsed LNPs. Hence, for the reduced rate of binding to be solely due to accumulation of negatively charged lipids near the site of LNP attachment, the relaxation of the LNP shape should be dramatic and engage the majority of charged MC3 lipids in an LNP (∼5.5 × 10^4^ MC3 lipids per LNP; see Supporting Information, section 2 for estimation). Of note, in experiments with more than 1 order of magnitude higher LNP concentration (∼25 × 10^9^ particles/mL) and a lower fraction of charged lipids η = 0.02 the LNP binding saturated at *C* = 0.3 μm^–2^ at pH 4.6 (Supporting Information, Figure S3) which is also far below the jamming limit of 100 μm^–2^ for LNPs with a radius of 40 nm. Under the assumption that the LNP collapse is dramatic and that a majority of MC3 (*n*_∗_ ∼ 5.5 × 10^4^) is accumulated in the contact zone, we have *c*_∗_ = *n*_∗_*C* = 2 × 10^4^ μm^–2^. This number is close to the average concentration *c* of charged lipids in the SLB at η= 0.02 (*c* = η/*s* = 2.4 × 10^4^ μm^–2^), which suggests that the pH-induced LNP attachment may indeed induce a dramatic collapse of the LNPs.

To scrutinize the diffusion of lipids to and out from the contact zone, it is instructive to first estimate the time scale, τ, corresponding to association of *n* negatively charged lipids to the LNP-SLB contact area. Roughly, the diffusion-limited flux *J* of these lipids is given by *J* ≈ *Dc*, and accordingly, τ = *n*/*J* ≈ *n*/*Dc*, where *D* = 3 μm^2^/s is the lipid diffusion coefficient. Setting *n* to 5.5 × 10^4^, i.e., engagement of the majority of the MC3 lipids, the time scale corresponding to lipid diffusion limitations τ becomes ∼0.2 s at *c* = 10^5^ μm^–2^. This is appreciably shorter than the time scale during which the rate of LNP binding diminishes at pH 5 and pH 4.6 (Figure 2), suggesting that if this hypothesis holds, the process is controlled by the time scale of LNP relaxation. This analysis also suggests that an additional contributing factor to the reduction in the rate of LNP binding may be attributed to escape of positively charged MC3 from collapsed LNPs into the SLB, being consistent with lipid transfer observed upon pH induced binding of MC3 containing LNPs to anionic lipid monolayers formed at the air water interface.^20^ Transfer of cationic lipids into the anionic lipid bilayer would lead to a reduction of net negative charge of the SLB, a process that was previously observed to influence electrostatically controlled liposome binding to oppositely charged SLBs.^52,53^

These findings are potentially relevant in the context of endosomal maturation arrest recently observed for endosomes containing multiple LNPs,^54^ since they suggest that both the charge balance between the total number of ionized MC3 and the limited number of negatively charged lipids available in the endosomal membrane as well as the time scale of LNP collapse may be crucial parameters for successful endosomal escape. Further, it is also worth noting that upon stepwise reversal of pH up to 7.5, ∼50% of the Cy5-labeled mRNA remained visible on the ncSLB, with most of the release events occurring when the pH was switched from pH 5.6 to 6.6 (Supporting Information, Figure S4). This observation further supports that a significant proportion of the LNPs undergo structural changes that are not reversed upon charge neutralization of MC3. It also shows that mRNA can be released upon increasing the pH above the isoelectric point of the LNPs, which is representative of mRNA coming in contact with the cellular cytosol after endosomal rupture.

**Figure 3:**
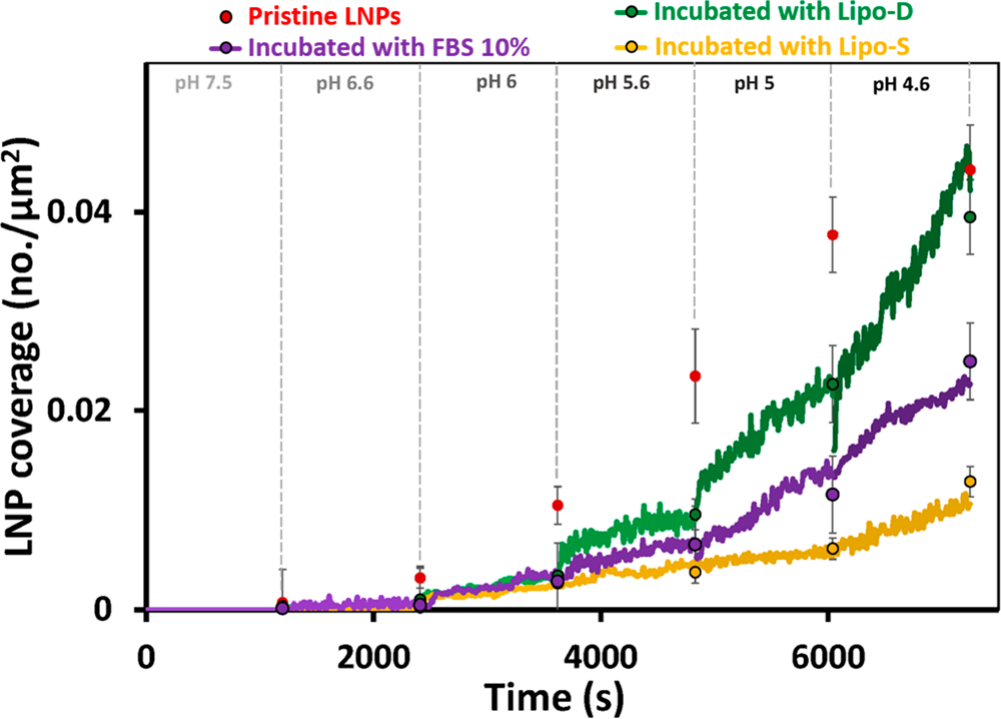
LNP adsorption kinetics extracted from experiments of the type shown in Figure 2: LNP coverage as a function of time at different pH and preincubation conditions for Lipo-D (green), FBS 10% (purple), and Lipo-S (gold). The averaged LNP coverage was obtained from single micrographs (*n* > 5) recorded just prior to the subsequent LNP injection at a lower pH and displayed as circles at the end of each step. Red circles correspond to the averaged coverage of pristine LNPs displayed here as reference. All samples were preincubated for 10 min with the indicated serum solutions.

The above-described function of pristine LNPs is of interest from a biophysics perspective but not fully representative in a biological context. In particular, LNPs are known to acquire protein coronas that are, in many cases, crucial to promote their cellular uptake. We therefore next asked whether such a corona would influence the magnitude and nature of the LNP interaction with the endosomal model membrane. To address this question, the experiments described above were repeated for LNPs that had been preincubated with serum-containing media prior to injection in the flow cell. Inspired by recent reports, demonstrating the important role of lipoprotein binding to LNPs for successful uptake and endosomal cargo escape,^12,13,55,56^ we specifically compared the pH-induced LNP binding to the endosomal membrane mimic for LNPs preincubated with diluted untreated fetal bovine serum (FBS 10%) with the corresponding binding patterns of LNPs preincubated with 10% lipoprotein saturated (Lipo-S) or lipoprotein-depleted (Lipo-D) fetal bovine serum (Figure 3). The relative presence of lipoproteins in the 10% FBS, Lipo-D and Lipo-S samples, obtained by LC/MS-MS, validated the depletion and enrichment of lipoproteins in the respective fractions (Supporting Information, Figure S5). Compared with pristine LNPs (Figure 3, red symbols), preincubation in 10% untreated FBS for 10 min prior to injection did not induce any detectable binding to the ncSLB above pH 6.6 (Figure 3, green). Instead, there was a significant shift in the onset of LNP binding to lower pH, and a more than 50% reduction in the rate of LNP binding at all pH conditions assayed. These results suggest that the FBS-induced protein corona formation on the surface of the LNPs, addressed in detail elsewhere,^14^ reduces the MC3-mediated pH-induced electrostatic attraction to the negatively charged SLB. However, there was no reduction in the rate of LNP binding even at the lowest pH (as observed for pristine LNPs, Figure 2), which suggests that protein corona formation at least in part prevents appreciable LNP collapse and lipid accumulation in the contact zone and/or exchange with the endosomal membrane mimic. We also found that the lipoprotein content of the FBS, and hence the formed protein corona, had significant effects on the interaction of the LNPs with the endosomal membrane mimic. Upon removal of lipoproteins from the preincubation serum by KBr-induced flotation (Lipo-D), the pH-induced LNP binding was still hampered, but eventually reached the same coverage as for pristine LNPs at pH 4.6. In contrast, for LNPs preincubated in lipoprotein supplemented serum (Lipo-S), the LNP binding was further reduced compared with the FBS 10%-treated sample at all pH. This suggests that lipoprotein association to LNPs, while reportedly often beneficial for cell uptake, may influence the crucial endosomal escape event.

**Figure 4:**
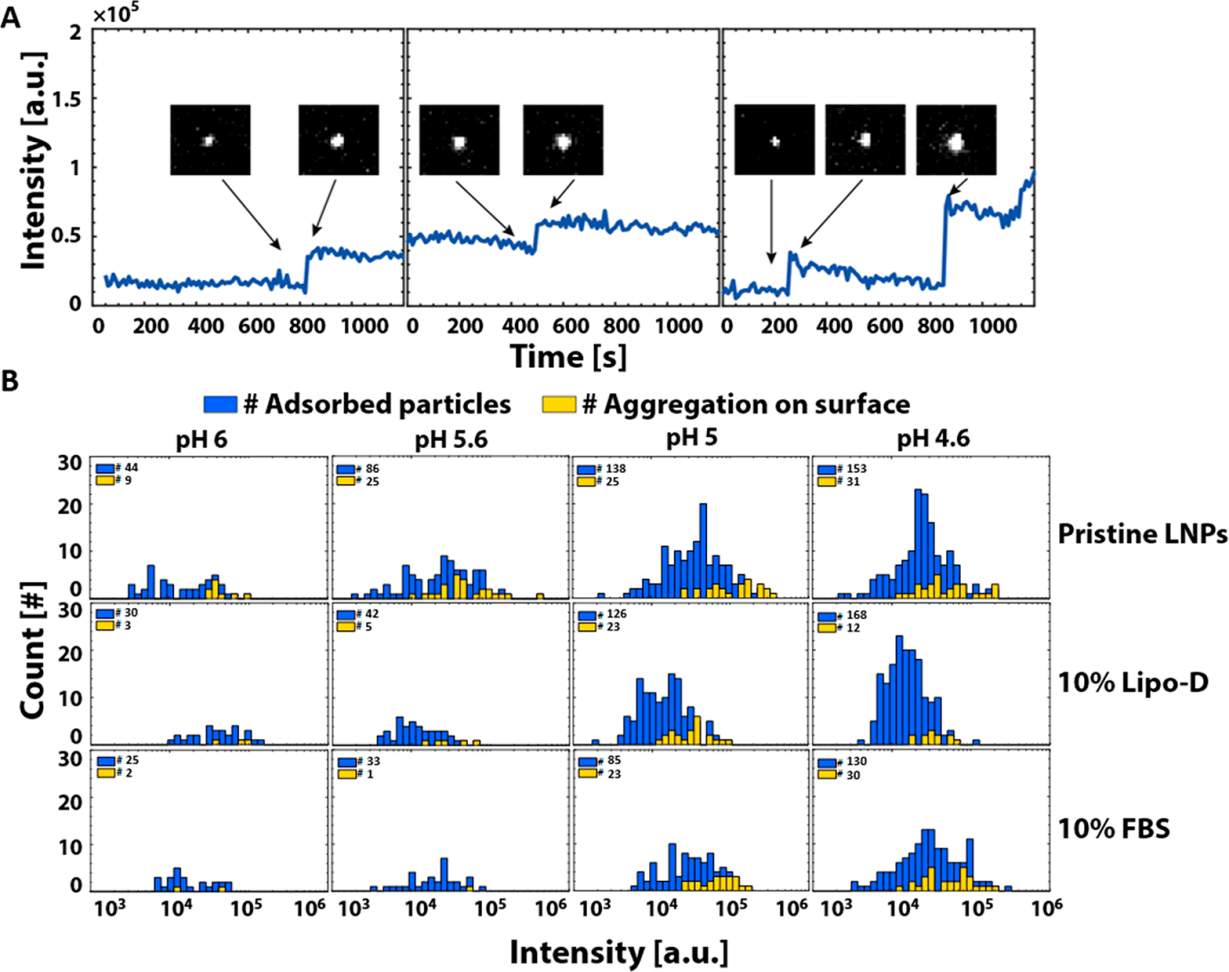
(A) Intensity profiles of three representative LNPs displaying sudden intensity increase after binding to the ncSLB (the insets show the corresponding TIRF micrographs (8.0 × 7.0 μm^2^) prior to and after the intensity increase. (B) Intensity histograms for all LNPs (blue) adsorbed to the ncSLB for pristine LNPs in comparison with LNPs preincubated in 10% Lipo-D and 10% FBS measured at the end of each experiment (∼1100 s). The yellow bars represent the fraction of LNPs that displayed one or more sudden intensity increases.

Aided by the single LNP resolution, the time lapse movies recorded during the TIRF experiments provided another curious observation: the bound LNPs displayed slow intensity variations (on the time scale of ∼200 s) which, for a significant fraction, was combined with one or sometimes two appreciable stepwise increases (on the time scale of ∼5 to 10 s) of the intensity at the locations of LNP binding (Figure 4A; Figure S6). Between ∼20 and 30% of the LNP binding events were accompanied by sudden intensity increases at pH 5.6 and below for pristine LNPs and at pH 5.0 and below for LNPs preincubation in serum (Figure 4B). Further, the intensity distribution at the end of each incubation ranged over more than 2 orders of magnitude, which is significantly broader than expected for LNPs with diameters ranging from 40 to 120 nm (Supporting Information, Figure S2). It is worth noting that there is a tendency that sudden intensity increase events are more likely to occur for LNPs with higher intensity. These observations suggest that an appreciable number of adsorbed LNPs undergo a significant collapse, since such events would transfer the fluorescent labels closer to the surface where the intensity of the evanescent field utilized in TIRF imaging is highest. This interpretation is further supported by a complementary experiment in which biotin-modified LNPs containing a small fraction of fluorescently labeled lipids were immobilized at neutral pH to a biotin-modified ncSLB using streptavidin as a linker. Upon reduction of pH, clear signatures of lipid transfer to the ncSLB, which is consistent with LNP collapse, was observed for a fraction of the LNPs (Supporting Information, Figure S7).

As detailed in section 8 of the Supporting Information, a complete LNP collapse would, for LNPs with diameters ranging from 40 to 120 nm, result in an intensity increase between 1.2 and 1.8, i.e., as observed, the effect is predicted to increase with increasing LNP size. This is in reasonable agreement with most events observed, which ranged between 1.1 and 2.5 (Figure 4A, left and central panels, and Figure S6A–G). Still some occasional events with significantly more pronounced intensity increases were also observed (Figure 4A, right panel and Figures S6H–J). This suggests that the interaction between the LNPs and the ncSLB may not only induce rapid collapse events, but also that later arriving LNPs can, in some instances, colocalize and aggregate with already bound LNPs. In principle, LNP aggregation might also occur in the bulk of the solution but this is unlikely because the interaction between LNPs is weak even in the absence of electrostatic repulsion (see section 9 of the Supporting Information for an estimate of the interaction strength between suspended LNPs). Thus, LNP-LNP aggregation is not expected to occur unless LNPs bound to the ncSLB would expose hydrophobic defects or negatively charged mRNA, which adds further support to a structural change of the LNP being induced when bound to the ncSLB. This interpretation is further supported by the fact that LNP collapse and/or aggregation was not observed at pH 6.6, displayed a peak at pH 5.6 for pristine LNPs and did not occur until pH 5.0 for LNPs preincubated in normal and lipoprotein-depleted serum (Figure 4B), where the protein corona may serve to reduce electrostatic attraction and possibly also to stabilize local defects on the surface of the LNPs. Further, mRNA was not observed to diffuse into the ncSLB, which is attributed to electrostatic attraction between the positively charged headgroup of MC3 and the negatively charged mRNA, which is expected to ensure firm association to the lipid membrane or the underlaying silica support.

These observations are curious in the context of the reported significance of nanoparticle clustering for modulating cellular uptake and endosomal escape.^57,58^ Further, even though lipoprotein depletion was not observed to influence the tendency of LNPs to undergo collapse and/or aggregation, lipoproteins seem to hamper the electrostatic attraction of LNPs to the endosomal membrane, which is, in turn, expected to drive the critical endosomal escape event. Since protein corona formation precedes and has even been shown to be required for efficient cellular uptake,^11−14^ these findings call for complementary live cell studies to gain deeper insight into the influence of protein corona formation in general, and lipoproteins in particular, not only on uptake efficiency, but also with respect to maintained coronation in the endosomal lumen and the impact of coronal proteins on endosomal escape.

**Figure 5:**
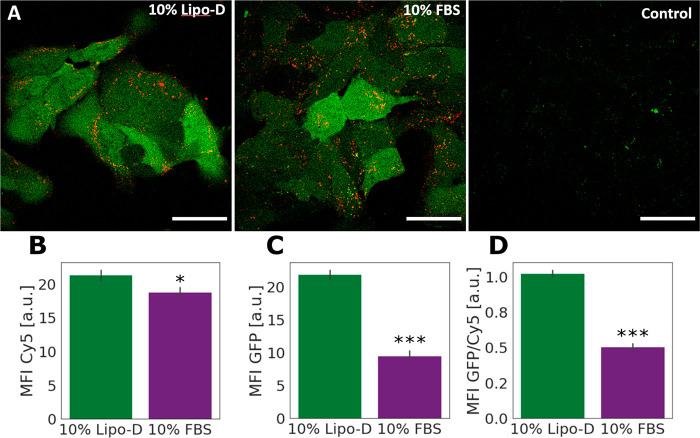
LNP uptake and eGFP expression in Huh7 cells exposed to LNPs freshly diluted in 10% Lipo-D or 10% FBS. (A) Representative confocal microscopy images of cells treated as mentioned above, including negative control (LNP-untreated cells). The images were acquired and treated with identical settings, and intensities are thus comparable. All scale bars are 50 μm. (B–D) Cell uptake (Cy5 signal, B), protein expression (eGFP signal, C), and endosomal escape efficiency (Cy5/eGFP signal, D), measured by flow cytometry (*N* = 3; error bars represent standard deviation of three independent biological replicates). The cells were incubated with LNPs at a mRNA concentration of 0.625 μg/mL for 2 h prior to analysis to allow for detectable levels of uptake and protein expression. Means comparison for data in B–D was performed by an unpaired Student’s *t* test, *N* = 3; * and *** denote statistically significant differences in mean at the *p* < 0.05 and *p* < 0.0001 levels, respectively. The *p* value for means comparison for the data in (B) was 0.037.

To investigate how the presence of lipoproteins in the preincubation serum influences the functional cellular delivery of the LNPs, we exposed cultures of hepatic Huh7 cells to MC3 LNPs containing mRNA encoding eGFP and monitored cell uptake and protein translation. Within a minute before cell exposure, the LNPs were diluted in cell culture media with 10% untreated FBS or 10% Lipo-D to represent lipoprotein-rich and -depleted protein coronas corresponding to the ones in the biophysical studies (Figures 2–4). The LNP uptake was visualized by Cy5 fluorescence from the labeled mRNA and functional delivery by the fluorescence of the expressed eGFP protein expression using confocal microscopy (Figure 5A) and quantified, after harvesting of the cells, with flow cytometry (Figure 5B–D). The results show that the uptake of the LNPs pretreated with FBS 10% and Lipo-D 10% (Figure 5A,B) is largely comparable (∼7% higher with Lipo-D). This indicates that a general depletion of lipoproteins in the serum does not prevent cellular uptake and is consistent with that the depletion method used only resulted in minor removal of ApoE (Supporting Information, Figure S5B), known to be crucial for cellular uptake.^10^ However, the cells that were treated with Lipo-D pretreated LNPs (and continuously kept in Lipo-D culture media throughout the experiments) displayed a significantly higher level of eGFP fluorescence (Figure 5C). Although protein expression is an aggregate readout that can be influenced by multiple processes, these results suggest improved delivery for LNPs preincubated in serum with lower lipoprotein content. Under the assumption that the endocytic mechanisms that control LNP uptake are not directly coupled to the mechanisms that dictate endosomal escape (pH-buffering effect, flip-flop mechanism, fusion or destabilization mechanism etc.),^59^ and the assumption that protein expression is not affected by the lipoprotein composition of the cell media, an indicative measure of relative endosomal escape efficiency can be obtained by normalizing the eGFP signal to the Cy5 signal (Figure 5D). The higher eGFP/Cy5 ratio for cells incubated in Lipo-D FBS compared to FBS suggests that endosomal escape could be promoted under lipoprotein-depleted conditions. This result agrees with the biophysical data (Figure 3), where LNPs preincubated with Lipo-D solution presented a more favorable interaction with the ncSLB.

## Conclusion

Time-resolved TIRF microscopy with single LNP resolution was used to provide biophysical insights with respect to how gradual acidification influences the interaction between ionizable lipid-containing LNPs and a supported lipid bilayer mimic of the endosomal membrane. Although the onset of LNP binding occurs at high pH (∼6.6), efficient binding does not occur until the pH is reduced to between 6.0 and 5.6, and appears to eventually be severely limited by suppression of the driving force for multivalent bond formation during LNP attachment or, more specifically, by accumulation of anionic lipids in the contact zone between the LNP and the membrane mimic accompanied by charge neutralization of anionic lipids in the model membrane due to their association with cationic lipids escaping from the earlier-attached LNPs upon their disintegration. This finding suggests that the nature of the interaction between LNPs and endosomal membranes may change significantly as LNPs mature along the endosomal pathway.

Aided by the single LNP resolution provided by TIRF imaging, we also conclude that a significant fraction of the LNPs undergo substantial stepwise collapse on the model membrane, a process that is likely a prerequisite for efficient cargo delivery across the endosomal membrane. The LNP collapse typically occurred within 5 to 15 min after initial LNP binding, being consistent with the previously observed lag phase associated with LNP-binding induced reorganization of anionic lipid monolayers formed at an air water interface.^20^ From a physical perspective, this is consistent with the relatively large length scale of the structural heterogeneity of the LNP, with a core composed primarily of mRNA and ionizable lipids surrounded by a shell composed of helper lipids, both of which only about three to four times smaller than the LNP size.^42,60^ Although a detailed mechanistic understanding of LNP collapse is lacking, our observations suggest that this process could potentially contribute to the endosomal escape and should therefore be considered when optimizing the design of LNPs containing pH-sensitive ionizable lipids. These insights may also be important to consider when evaluating endosomal escape capacity since single endosomes are likely to contain multiple LNPs and since the total number of negatively charged lipids available in a maturing endosome may be a limiting factor for attachment, fusion, and escape. A relevant extension of the work is therefore to investigate LNPs made using different ionizable lipids as well as different lipid compositions and cargo content.

We also observed that preincubation of the LNPs in serum solutions shifts the onset of LNP binding toward lower pH, suggesting that proteins adsorbed on the surface of the LNP may hamper the interaction between LNPs and the endosomal membrane. This effect might actually influence the endosomal escape efficiency, making this observation interesting in the context of previous studies demonstrating particularly productive endosomal escape early in the endolysosomal pathway.^61,62^ Further, preincubation of LNPs in lipoprotein-depleted (Lipo-D) serum caused a weaker inhibitory effect on the electrostatic attraction between the LNPs and the supported endosomal model membrane. We also observed more protein (eGFP) translation in live cells treated with Lipo-D preincubated LNPs compared to the FBS 10% case. Although not possible to draw firm conclusions regarding endosomal escape from differences in the ratio between LNP uptake and protein production, these results suggest that although binding of lipoproteins to the surface of LNPs has been identified as crucial for efficient cellular uptake,^12,13^ it may be equally important to consider how this class of proteins may impact the efficiency of the actual mRNA endosomal escape event as well as other mechanisms that control protein production.

Although further studies are required to verify the biological significance of the biophysical insights gained using a reductionist mimic of the endosomal membrane as done in this work, the approach is readily transferable to a wide range of drug-delivery vehicles and more realistic model membrane systems,^63,64^ from which insights could be gained that should aid the design of next generation lipid-based nanoparticles and more efficient oligonucleotide delivery.

## Materials and Methods

### Lipid Nanoparticle Fabrication

We used in this work mRNA containing LNP batches with good in vitro transfecting efficiency for human adipocytes and hepatocytes.^42^ The LNP contained the ionizable cationic lipid *O*-(*Z*,*Z*,*Z*,*Z*-heptatriaconta-6,9,26,29-tetraem-19-yl)-4-(*N*,*N*-dimethylamino)butanoate (DLin-MC3-DMA), 1,2-distearoyl-*sn*-glycero-3-phosphocholine (DSPC), cholesterol (Chol), and 1,2-dimyristoyl-*sn*-glycero-3-phosphoethanolamine-*N*-[methoxy(polyethylene glycol)-2000] (DMPE-PEG2000) (structures of all lipids are shown in Figure S2A). DLin-MC3-DMA contains an ionizable amino group which obtains a positive charge at low pH via protonation of the amine moieties (Figure S2A), which together with the hydrophobic tails facilitate self-assembly with the other lipids and encapsulation of the mRNA payload into nanoparticles due to electrostatic attraction between ionized MC3 and the anionic nucleic acids. The LNPs were prepared using the NanoAssemblr Benchtop device (Precision Nanosystems Inc., Canada). Briefly, stocks of MC3, DSPC, Chol, and DMPE-PEG2000 lipids were dissolved in ethanol and mixed in a mol % ratio of 50:10:38.5:1.5 to obtain a lipid concentration of 12.5 mM (1.85 mg/mL). The mRNA solution was prepared by mixing CleanCap cyanine5 EGFP mRNA and CleanCap EGFP mRNA (1 mg/mL, Trilink Biotechnology) in a 1:5 volume ratio, then diluted in 50 mM RNase-free citrate buffer pH 3.0 to a concentration of 0.25 mg/mL. The mRNA and lipid solutions were mixed in a 3:1 volume ratio through a microfluidic cartridge of the benchtop device at a flow rate of 12 mL/min to obtain a mRNA:lipid weight ratio of 10:1 (molar ratio nucleotide:MC3 of 1:3.08) in the final LNP formulation. LNPs were dialyzed overnight against 600× sample volume using Slide-A-Lyzer G2 dialysis cassettes from Thermo Scientific with a molecular weight cutoff of 10 K. The collected LNPs were filtered through a sterile filter (0.2 μm) prior any measurement. The LNPs were characterized with dynamic light scattering (DLS) to the determine size, concentration, and polydispersity index (PDI), and with RiboGreen assay to obtain the encapsulation efficiency (EE) and mRNA concentration. Measurements showed that the LNP batches used in this work contained 0.134 mg/mL of mRNA (97% EE), had an average diameter of 78 nm, were highly monodispersed (PDI = 0.085), and contained 1.3 × 10^13^ LNP particles per milliliter (Figure Sd2B).

### TNS Assay

The anionic fluorescent dye 2-(*p*-toluidino)-6-napthalene sulfonic acid measurements were performed in a 384-well format with a buffer containing 20 mM phosphate tribasic, 25 mM ammonium citrate, 20 mM ammonium acetate, and 150 mM sodium chloride, with a pH ranging from 3 to 11. The molar ratio of total lipid:TNS dye was kept at 4.25, and the total lipid concentration in each well was kept at 7.3 μM. All measurements were performed at room temperature within 10 min of preparation using a fluorescence plate reader (BMG Labtech) with excitation at 340 nm and emission at 460 nm.

### Endosomal Model System

Negatively charged synthetic vesicles (ζ = −22.6 ± 2.05 mV obtained by DLS) were formulated in phosphate-buffered saline (PBS) using the lipid film hydration and extrusion method^65^ and were used to form supported anionic model membranes. A citrate–phosphate buffer was used to adjust the pH in the range of 7.5 to 4.6. The lipid materials, 16:0–18:1 PC:1-palmitoyl-2-oleoyl-*sn*-glycero-3-phosphocholine (POPC), 16:0–18:1 PS:1-palmitoyl-2-oleoyl-*sn*-glycero-3-phospho-l-serine (POPS), 18:1 NBD PE:1,2-dioleoyl-*sn*-glycero-3-phosphoethanolamine-*N*-(7-nitro-2–1,3-benzoxadiazol-4-yl) (NBD), were purchased from Avanti Polar Lipids, Inc. in liquid form (in chloroform) with the concentration of 10, 10, and 1 mg/mL, respectively. To formulate the vesicles, 186.45 μL of POPC (93.5 mol %), 12.34 μL of POPS (6 mol %), and 12.12 μL of NBD (0.5 mol %) were mixed and dried in vacuum overnight. The lipid film was rehydrated with PBS for 1 h to a total lipid concentration of 2 mg/mL. The solution was then subsequently extruded 21 times using a mini extruder (Avanti Lipids Inc., Alabaster, AL, USA) with 50 and 30 nm polycarbonate membranes (Whatman, Maidstone, UK) to form the vesicles with the size of approximately 80 nm. The vesicle solution was stored at 4 °C for later use.

To prepare the microfluidic channel borosilicate cover glasses (Menzel-Gläser, D263, number 1) were first submerged in ETOH–NaOH (5:1) cleaning solution for 5 min and rinsed thoroughly with deionized water (Milli-Q, Merck Millipore), after which they were dried by nitrogen and treated with UV-ozone for 20 min followed by second Milli-Q rinsing step, nitrogen drying, and 20 min UV-ozone treatment before being assembled with the Ibidi sticky microfluidic channels (3.8 × 17 × 0.4 mm in *w* × *l* × *h* and 30 μL of volume in the channel; #80608, Ibidi cell in focus, Gräfelfing, Germany).

A negatively charged SLB was formed on the floor of the microchannel by injection of a lipid vesicle suspension (diluted in the PBS buffer to a lipid concentration of 200 μg/mL), resulting in spontaneous formation of a continues and homogeneous SLB^65^ as verified using FRAP assessment as described together with the TIRF imaging setup below.

### Serum and Protein Preparation

Lipoproteins were separated from FBS using Havel’s method.^66^ The density of FBS was increased to 1.21 g/mL by addition of KBr, after which it was ultracentrifuged at 55,000 rpm at 10 °C for 14 to 24 h (overnight) using a Beckman Optima XL-100 K and a type 90 Ti rotor. The upper lipoprotein-saturated (Lipo-S; ∼1 mL) and the lower lipoprotein-depleted (Lipo-D; ∼8 mL) fractions were separated by transferring into separate tubes for dialysis (Figure S5A). Dialysis of both fractions to remove KBr was performed using dialysis cassettes with a 2000 kDa membrane, using 1.3 l PBS for every 7 mL sample for 4 h at 4 °C, repeated twice, and followed by an overnight dialysis step for complete removal of KBr. Finally, the samples were pushed through a 0.2 μm filter for purification. The proteomics data of the FBS 10% with the Lipo-D and Lipo-S samples were obtained by LC/MS-MS to compare the lipoprotein concentrations (Figure S5B).

### TIRF Microscopy

The microfluidic system was mounted on an inverted Eclipse Ti-E microscope (Nikon Corporation, Minato City, Japan) equipped with a CFI Apo TIRF 100× (NA: 1.49) oil immersion objective (Nikon Corporation, Tokyo, Japan) for recording time-lapsed movies. A FITC filter set (Semrock, Sandwich, IL, USA) was used for visualizing the vesicles adsorption and subsequent supported lipid bilayer formation on the glass floor of the channel. In addition, the ncSLB formation and quality of bilayer were validated using FRAP by bleaching NBD lipids in a circular region (spot) of the bilayer with a Kr–Ar mixed gas ion laser (Stabilite 2018, Spectra-Physics Lasers, Mountain View, CA, USA) at a wavelength of 531 nm followed by imaging of the fluorescent recovery with 5 s interval. The diffusivity of lipids within the bilayer was acquired, as a signature of bilayer qualities, by analyzing the recovery data using a custom written code in MATLAB R2016B V9.1 (MathWorks. Inc., USA).^47^

To quantify the interaction of LNPs preincubated in 10% FBS, Lipo-D, or Lipo-S with endosomal model membranes, 1 μL of LNPs were incubated with 7 μL of the serum sample at room temperature for 10 min prior to the experiments.

To visualize the LNPs, while maintaining their functionality, 20% of the Cy5-mRNA were incorporated in the cargo (0.01 fg of in total 0.06 fg of mRNA per LNP). LNPs were further diluted to a final concentration of ∼0.7 × 10^9^ particles/mL (3000× dilution) to facilitate the single-particle resolution prior to the injection of the LNPs into the microfluidic channel (using a syringe pump at withdraw mode, flow rate of 150 μL/min, and room temperature). Particles were diluted and preincubated in phosphate-citrate buffer at the desired pH for 5 min before the experiment to ensure the protonation. LNPs interaction with the ncSLB was monitored with a TIRF microscope using a Cy5 ET filter set (F46-006 ET-set, Chroma Technology Corporation, USA), where consecutive LNP injections at constant concentration and reducing pH values (6.6, 6.0, 5.6, 5.0, and 4,6) were imaged in time-lapse mode (6 frames per minute, 500 ms exposure) for a minimum of 20 min, with snapshot images taken at multiple parts of the SLB prior to each injection. The number of adsorbed particles per frame was counted to obtain the surface coverage (normalized to the field of view area: 6710.9 μm^2^) over time per sample and pH using ImageJ and a custom written code in MATLAB R2016B V9.1.

### Cell Culture

Human hepatic Huh-7 cells (kind gift from Prof. Samir El-Andaloussi, Karolinska Institute) were cultured in cell culture media (CCM) containing DMEM high glucose, 2 mM l-glutamine, 1 mM sodium pyruvate, and 10% FBS. The cells were dissociated and passaged using calcium/magnesium-free DPBS and Trypsin-0.25% EDTA. The medium was exchanged every 3 days during cultivation. The cells were tested and verified mycoplasma-free.

### Flow Cytometry

Flow cytometry was used to quantify LNP uptake (here using a Cy5-labeled mRNA) and measure the expression levels of mRNA through the fluorescence of the expressed eGFP reporter protein. Huh-7 cells were seeded in 48-well plates, at a density of 45,000 cells per well in 250 μL of complete media, 1 day before exposure. LNPs were diluted to at a final concentration of 0.625 μg/mL in CCM with the addition of either FBS 10% or Lipo-D and exposed to cells immediately upon dilution. After 2 h exposure, the treatment solutions were removed, and cells were washed twice with PBS and harvested using trypsin for 10 min at 37 °C. After detachment an equivalent volume of CCM was added, and all samples were transferred to a 96-well round-bottom plate and analyzed on a Guava easyCyteTM 8HT from Millipore. eGFP was excited at 488 nm and emission collected at 525/30 nm, and Cy5 was excited at 635 nm and emission collected at 661/15 nm. All experiments were carried out in triplicate. Cytotoxic effects were observed for the cells exposed to CCM with the addition of Lipo-S fraction, and the data collected for this condition were therefore not analyzed further.

### Confocal Microscopy

Confocal microscopy was used to visualize the distribution of LNPs in cells and the expression of the mRNA-encoded eGFP protein. Huh-7 cells were seeded in 4-sectors subdivided CELLview dishes at a density of 45,000 cells per chamber in 250 μL of complete media, 1 day before exposure. LNPs were diluted to at a final concentration of 0.625 μg/mL in CCM with the addition of either FBS 10% or Lipo-D and immediately added to cells. After 2 h exposure, the cells were washed with fresh CCM and imaged on a Nikon C2+ confocal microscope equipped with a C2-DUVB GaAsP detector unit and using an oil-immersion 60× 1.4 Nikon APO objective (Nikon Instruments, Amsterdam, The Netherlands). The 488 nm laser line was used to excite eGFP, and the emission detected at 496–566 nm, and Cy5 was excited at 640 nm and the emission detected at 652–700 nm. Identical settings were applied for all different conditions. The images were processed with Fiji ImageJ software.

## Supporting Information Available

Additional materials and methods including information about LNP preparation and characterization, serum sample preparation and validation, LNP binding kinetics as well as theories and additional experiments to support the LNPs collapse upon binding (PDF)
Movie S1: Binding of LNPs preincubated at pH 4.6 to ncSLB at pH 7.5 (AVI)

## Supplementary Material

nn2c04829_si_001.pdf

nn2c04829_si_002.avi

## Abbreviations

LNP: lipid nanoparticle
eGFP: enhanced green fluorescent protein
Cy5: cyanine 5
mRNA: messenger ribonucleic acid
DLS: dynamic light scattering
NTA: nanoparticle tracking analysis
TIRF: total internal reflection fluorescence
PEG: poly(ethylene) glycol
Dlin-MC3-DMA: *O*-(*Z*,*Z*,*Z*,*Z*-heptatriaconta-6,9,26,29-tetraen-19-yl)-4-(*N*,*N*-dimethylamino)
DSPC: 1,2-distearoyl-*sn*-glycero-3-phosphocholine
DMPE: 1,2-bis(dimethylphosphino)ethane
FBS: fetal bovine serum
CCM: cell culture medium
DTT: dithiothreitol
ncSLB: negatively charged supported lipid bilayer
Lipo-D: lipoprotein-depleted
Lipo-S: lipoprotein saturated
TNS: 2-(*p*-toluidino)-6-napthalene sulfonic acid
