# Supplementary material for: Interaction Kinetics of Individual mRNA-Containing Lipid Nanoparticles with an Endosomal Membrane Mimic: Dependence on pH, Protein Corona Formation, and Lipoprotein Depletion
Source: ACS Nano. 2022 Dec 13;16(12):20163–73. doi: 10.1021/acsnano.2c04829 (PMC9798854; doi:10.1021/acsnano.2c04829)
Supplement: Supplementary file 1 — nn2c04829_si_001.pdf [file nn2c04829_si_001.pdf]

SUPPORTING INFORMATION

Interaction Kinetics of Individual mRNA-Containing Lipid Nanoparticles with an Endosomal Membrane Mimic: Dependence on pH, Protein Corona Formation, and Lipoprotein Depletion

Nima Aliakbarinodehi[1], Audrey Gallud[2,3], Mokhtar Mapar[1†], Emelie Wesén[2], Sahar Heydari[2], Yujia Jing[3], Gustav Emilsson[3], Kai Liu[3], Alan Sabirsh[3], Vladimir P. Zhdanov[1,4], Lennart Lindfors[3], Elin K. Esbjörner[2], and Fredrik Höök[1]*

1) Division of Nano and Biophysics, Department of Physics, Chalmers University of Technology, 41296 Göteborg, Sweden

2) Division of Chemical Biology, Department of Biology and Biological Engineering, Chalmers University of Technology, 41296 Göteborg, Sweden

3) Advanced Drug Delivery, Pharmaceutical Sciences, R&D, AstraZeneca, 43181 Gothenburg, Sweden

4) Boreskov Institute of Catalysis, Russian Academy of Sciences, Novosibirsk 630090, Russia

KEYWORDS: ionizable lipid nanoparticle, mRNA delivery, endosomal membrane, protein corona, lipoprotein

Table of Content

1. FRAP assessment at various pH levels 2
2. LNP preparation and characterization 3
3. Saturation of LNP binding to ncSLB (with 2% negatively charged lipid) at pH 4.6 5
4. Protonation of MC3 lipid and the pH-sensitive interaction of LNPs with ncSLB 5
5. Lipoprotein depleted serum preparation and validation 6
6. Typical kinetics for increment of the LNP local intensity on the surface 7
7. Characterization of lipid translocation to an ncSLB upon pH-induced LNP collapse 8
8. TIRF intensity of attached LNPs 9
9. Interaction between LNPs in solution 11
10. Specifics of measurements under flow conditions 14
11. References 15

1. FRAP assessment at various pH levels

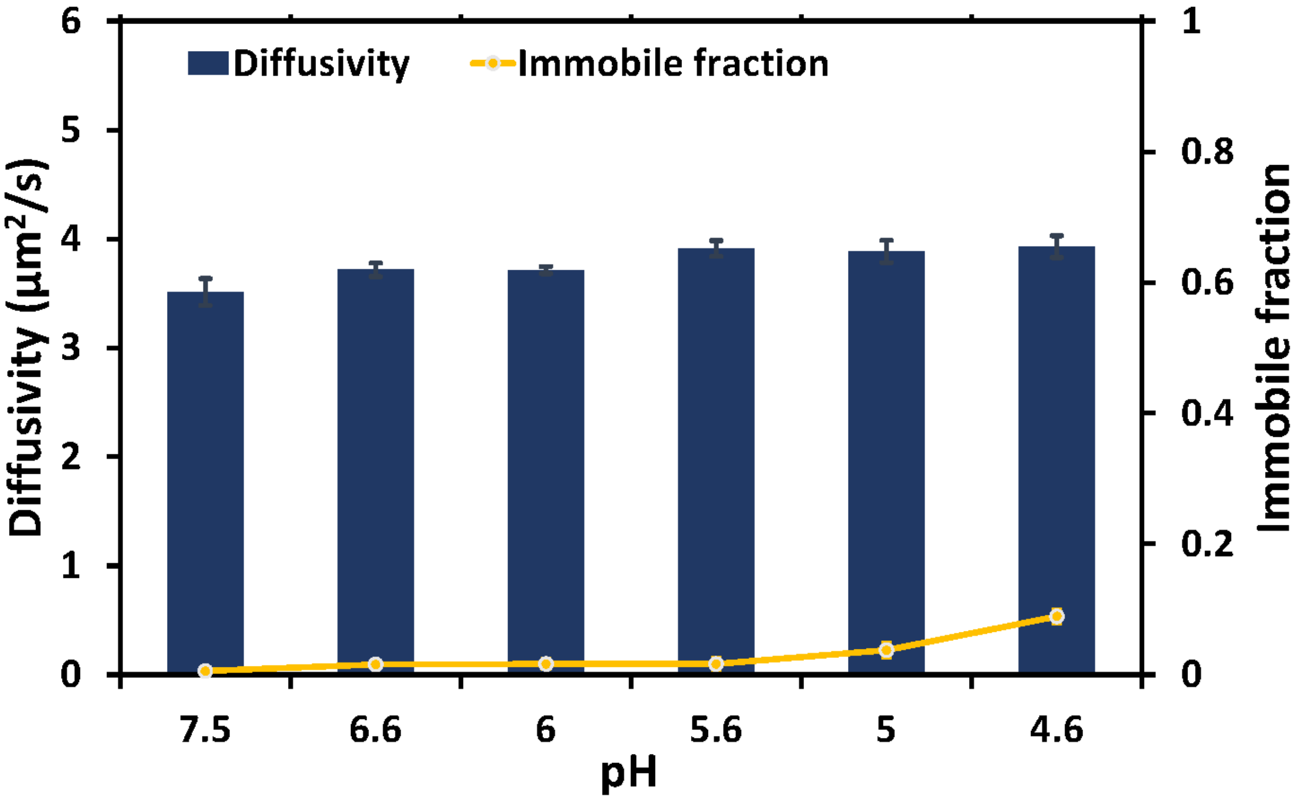

Figure S1. FRAP analysis performed on the ncSLB at the various pH employed in the assay. The data showed a stable diffusivity throughout the experiment with a negligible increase in the immobile fraction of the lipids.

2. LNP preparation and characterization

The structures of the lipids used for the LNP formation (see Materials and Methods in Main text) are shown in Figure S2A together with size and concentration determination of the LNPs after 50 times dilution in PBS using DLS measurements (ZetaSizer Nano ZS from Malvern Instruments Ltd) to be 78 nm and ~1.3×10^{13} particle/ml), respectively (Figure S2B). The volume-weighted particle size distributions were calculated using a particle refractive index of 1.45. The encapsulation and concentration of mRNA were determined using the RiboGreen assay.

The number of MC3 lipids per LNPs was determined from the final 3.08 molar ratio of the negative to positive charge in the formulation solution, resulting in an effective molar ratio of 3.17 considering an encapsulation efficiency of 97%. This results in $(0.134\ \mathrm{mg/mL})/(363\ \mathrm{g/mol} \times 3.17) = 1.17$ mM of MC3 or 7.1×10^{17} MC3 molecules/mL. Considering the LNP concentration (1.3×10^{13} LNP/mL) we obtain an average number of ~55000 MC3 molecules/particle.

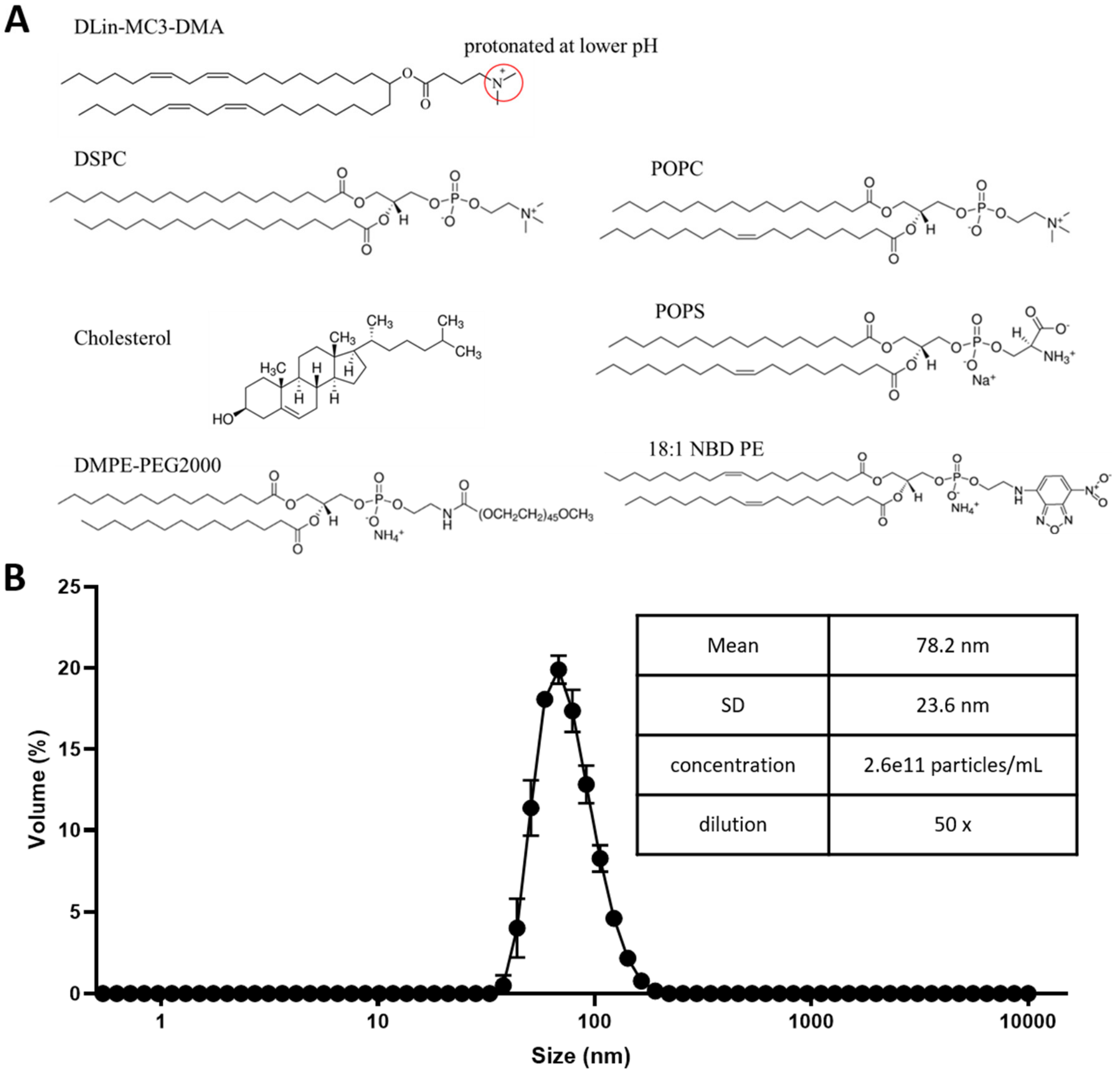

Figure S2. A) Structures of lipids used in this study; B) Average size distribution of LNP measured by DLS. Inset shows the measured size (volume-weighted based) and concentration of the LNPs after 50 times dilution.

3. Saturation of LNP binding to ncSLB (with 2% negatively charged lipid) at pH 4.6

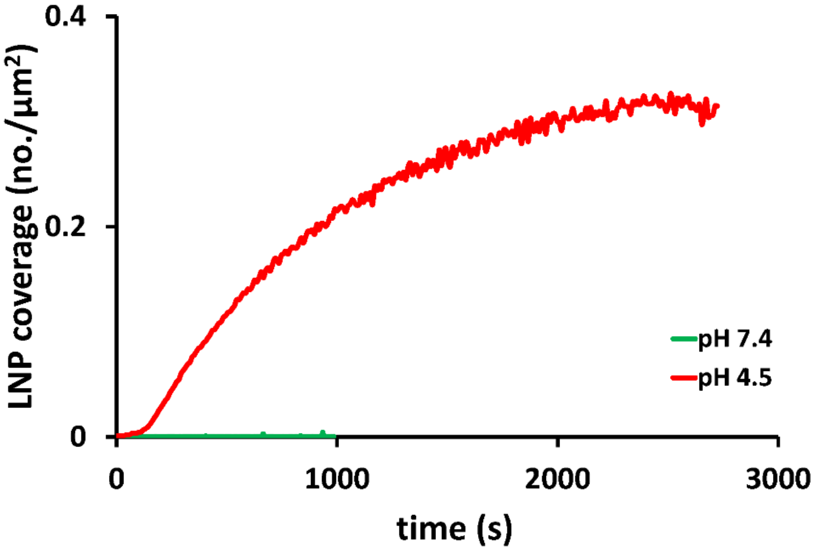

Figure S3. Adsorption of the pristine LNPs (~25×10^9 particles/ml) to the ncSLB with 2% of negatively charged lipid (z = -15.6 ± 1.02 mV). Curves show the saturation coverage and saturation time that are relative to the negative potential of the SLB and concentration of the LNPs.

---

4. Protonation of MC3 lipid and the pH-sensitive interaction of LNPs with ncSLB

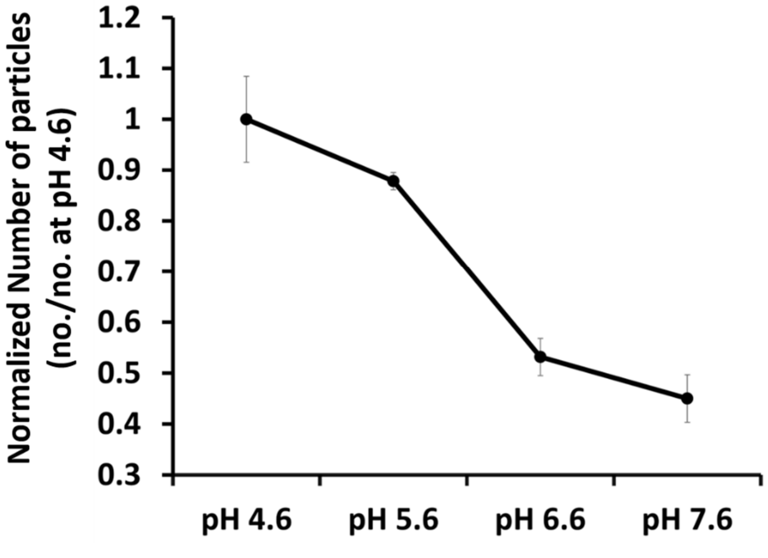

Figure S4. Identifying the role of MC3 cationic ionizable lipid in LNPs electrostatic adsorption by binding them at pH 4.6 followed by stepwise pH increment. 12%, 35% and 8% release of bound LNPs from the surface was observed for subsequent increasing of the pH from 4,6 to 5.6, 6.6, and 7.6. Majority of desorption happened at the increment from pH 5.6 to 6.6 (64% of the total

desorption). This range coincides with the apparent pKa of MC3 LNPs that is reported to be 6.4,[1] which is also what was determined in this study (see Main text).

5. Lipoprotein depleted serum preparation and validation

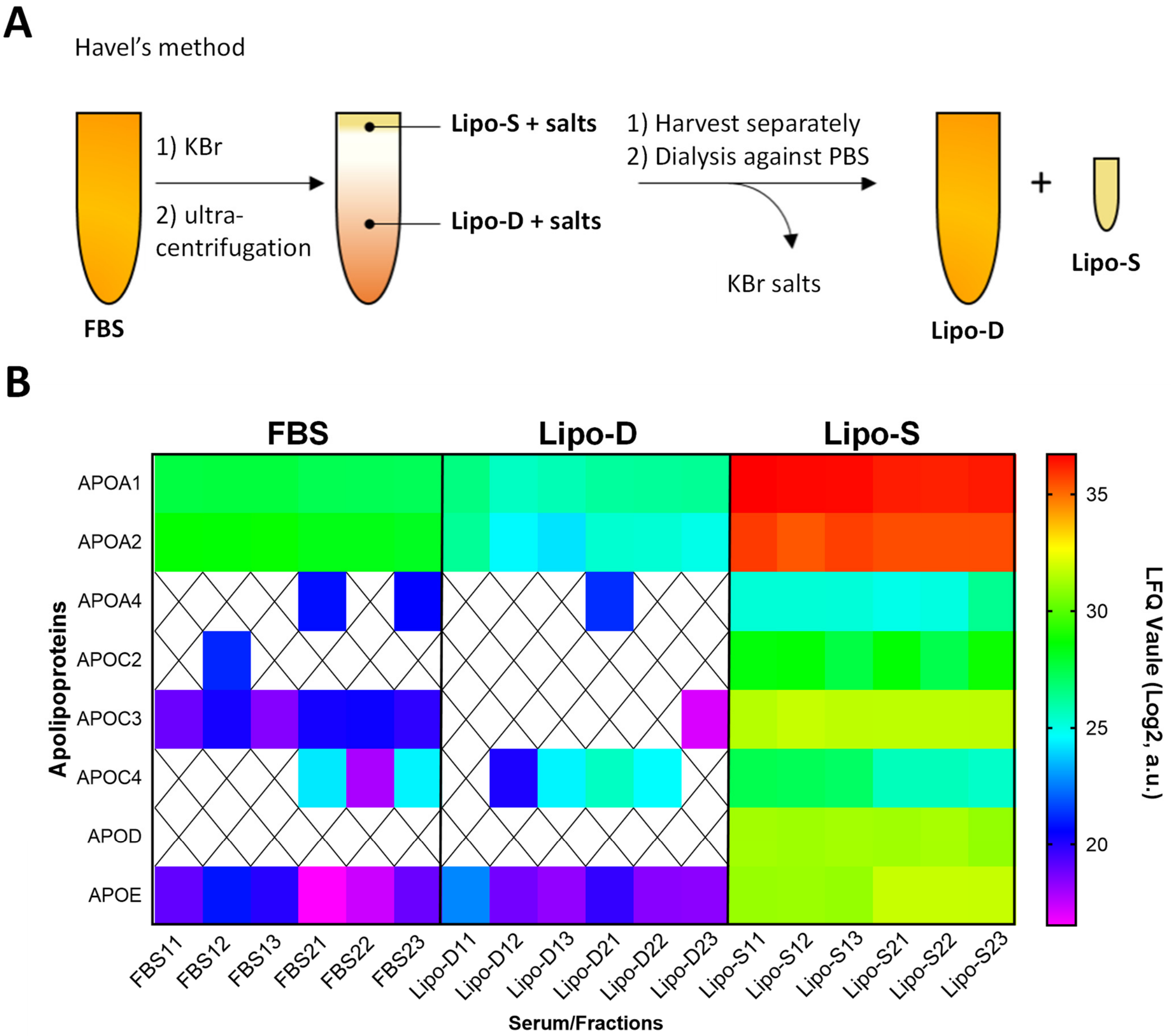

Figure S5. A) To investigate the role of serum lipoproteins for LNP uptake and mRNA release efficiency, cell culture medium with different lipoprotein contents were prepared using Havel's methods.[2] B) Indirect validation of apolipoprotein depletion by LC/MS-MS. The color bar presents the concentration level. Areas without color has concentration below detection limit. LFQ: label-free quantification.

6. Typical kinetics for increment of the LNP local intensity on the surface

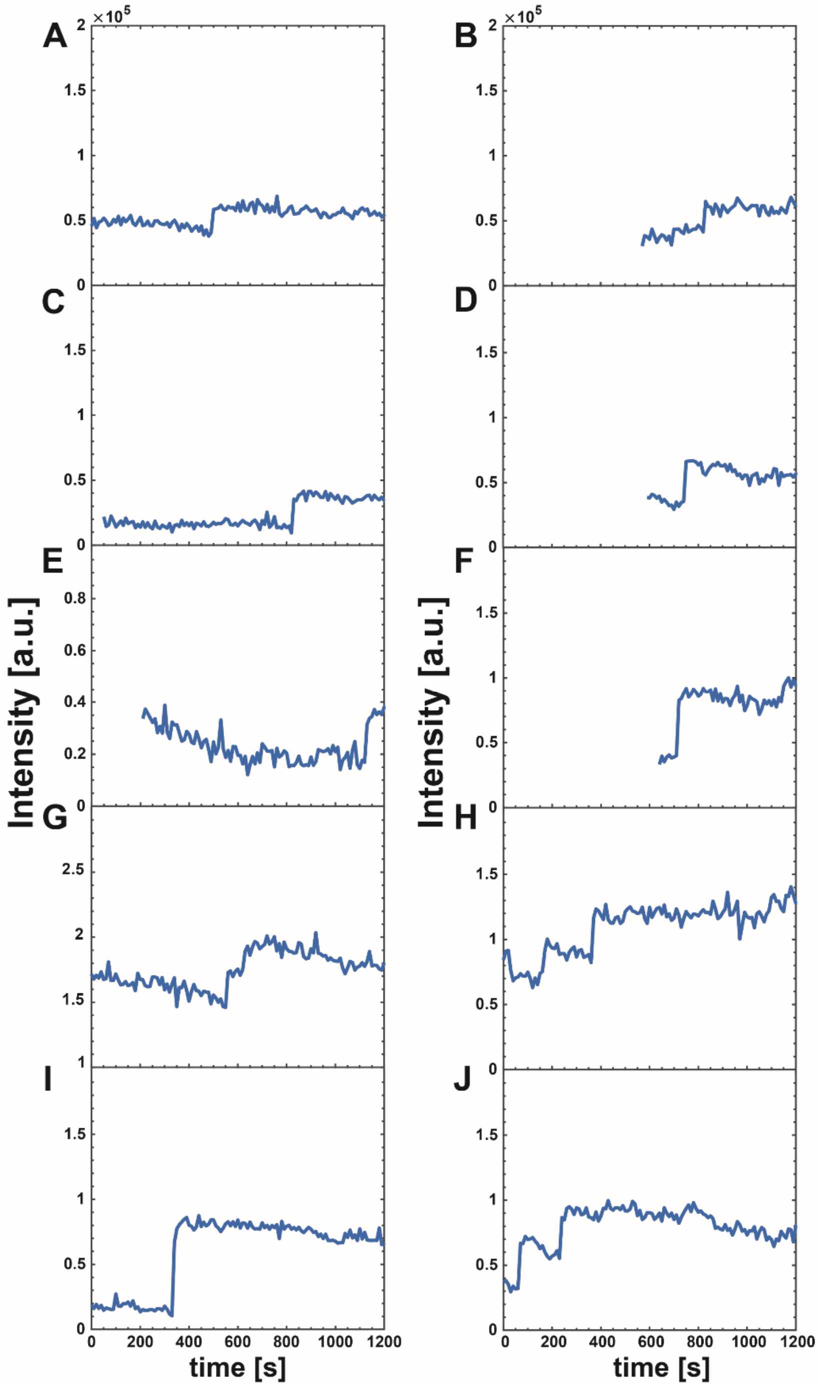

Figure S6. Typical temporal behavior of LNP local intensities on the surface, over time, following the acidification of environment and pH-induced electrostatic bindings. While kinetics with one step signal increase were abundant, two step increments were rarely observed. Note that the stepwise increase of the intensity is typically in the range 1.1 to 2.5 (e.g., panels A to G) and rarely

appreciably larger (e.g., panels H to J). We associate the former stepwise events with LNP collapse whereas the latter events with LNP aggregation.

7. Characterization of lipid translocation to an ncSLB upon pH-induced LNP collapse

To investigate the nature of the pH-induced interaction between LNPs and a negatively charged SLB in further detail, LNPs containing 0.06 mole% rhodamine labeled DOPE lipids were prepared as described in the Main text, but with slightly altered lipid composition (see the caption of Figure S7) to obtain LNPs with a mean diameter of ~130 nm,[3] which in turn facilitates fluorescence imaging without having to include a high concentration of fluorescently labeled lipids. In analogy with previous TIRF-based inspection of LNPs bound to a streptavidin modified glass surface,[4] the presence of biotin-PEG_{2000}-DOPE enabled streptavidin mediated LNP binding at pH 7.5 to biotin-modified ncSLB of the same type as used in the Main text (Figure S7A). After LNP binding to the streptavidin modified ncSLB, the pH was subsequently lowered to 5.6. Upon pH reduction, a fraction of LNPs displayed a rapid (sub-second) increase in fluorescence followed by an exponential decrease with a rate constant of around 2 seconds (Figure S7B). The initial increase in the fluorescence intensity is attributed to translocation of fluorescent compounds to the interface, at which the excitation intensity of the TIR illumination has its maximum. The subsequent decrease in the fluorescent signal is interpreted as escape of fluorescently labeled lipids into the ncSLB, being consistent with lipid exchange between the LNP and the ncSLB. These features are both consistent with the step wise collapse observed in the Main text.

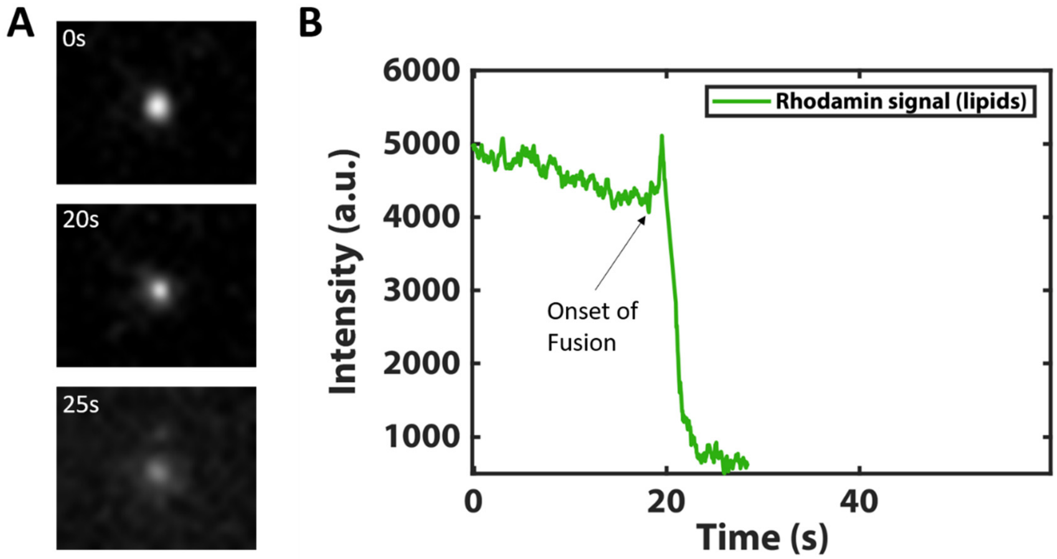

Figure S7. A) Micrograph snapshot (image size: 6.6×4.6 μm) of LNPs (MC3, DSPC, Chol and DMPE-PEG2000 lipids in mol% ratio of 53.47:4.65:41.114:0.7 and containing also 0.06% rhodamine-labeled DOPE and 0.006% DSPE-PEG(2000) biotin) immobilized using streptavidin to a biotin-modified SLB (POPC:POPS:biotinyl-CAP-PE:NBD-PE, with a molar ratio 93.45: 6:0.05:0.5) at pH 7 visualized in time resolved TIRF (acquisition rate: 250 ms) prior to and after changing the buffer to pH 5.6. B) Time resolved changes in the fluorescence intensity for one of the LNPs that display lipid release.

---

8. TIRF intensity of attached LNPs

The TIRF intensity of an attached LNP depends on its size and shape and the decay length, δ, of the intensity (square of the amplitude) of the exponentially vanishing evanescent field and can be represented as

$$I = AJ_0 v_c c_d \varphi, \quad (1)$$

where J_0 is the intensity of the incident light, v_c is the core volume (i.e., a part of the LNP volume containing dye), c_d is the dye concentration in the core, $\varphi \leq 1$ is the factor taking the evanescent field into account, and A is the sensitivity factor. The LNP size (diameter) in the intact state and δ are comparable, and accordingly before collapse the reduction of the TIRF intensity due to the

extinction of the evanescent field, i.e., since $\varphi < 1$, is not negligible provided the LNP deformation is modest. Upon LNP collapse, it is reasonable to consider that the dye molecules are integrated with the SLB, and accordingly φ is close to unity. Thus, the increase of the TIRF intensity upon collapse is equal $1/\varphi$, where φ is the factor corresponding to the LNP state before collapse. The corresponding expression for φ was obtained in ref. 5 (Eq. 10 there) in the context of localized surface plasmin resonance in the case when a spherical particle directly contacts the sensor surface. In that case, φ is a function of the ratio of the particle radius, r, and δ, i.e., $\varphi = F\,(r/\delta)$ (Eqs. 10-12 in ref. 5). In our case, an LNP and the sensor surface are separated by an SLB with thickness l, and the dye molecules are located within the LNP core. The core and LNP radii are related as $r_c = r_{LNP} - l$, where l is the lipid-bilayer thickness (as in the case of SLB). Thus, we have

$$\varphi = \exp\left(-\frac{2l}{\delta}\right) F\left[\frac{r_{LNP}-l}{\delta}\right], \tag{2}$$

where $\exp(-2l/\delta)$ is the factor taking the distance between the core and the sensor surface into account, and $F[(r_{LNP} - l)/\delta]$ is the factor related to the evanescence field inside an LNP. Our calculations based on Equation 2 indicate that for LNPs of the size we use (with $20 \leq r_{LNP} \leq 60$ nm) φ is in the range from $\simeq$0.8 down to $\simeq$0.55 (Figure S8) and accordingly the factor $1/\varphi$ characterizing the increase of the LNP TIRF intensity upon collapse is in the range from $\simeq$1.3 up to $\simeq$1.8.

These calculations do not take into account that the dye population in LNPs of the same size can be slightly different due to fluctuations which are inevitable during fabrication of LNPs. Due to these fluctuations, the distribution of the LNP TIRF intensity can be slightly wider than that estimated.

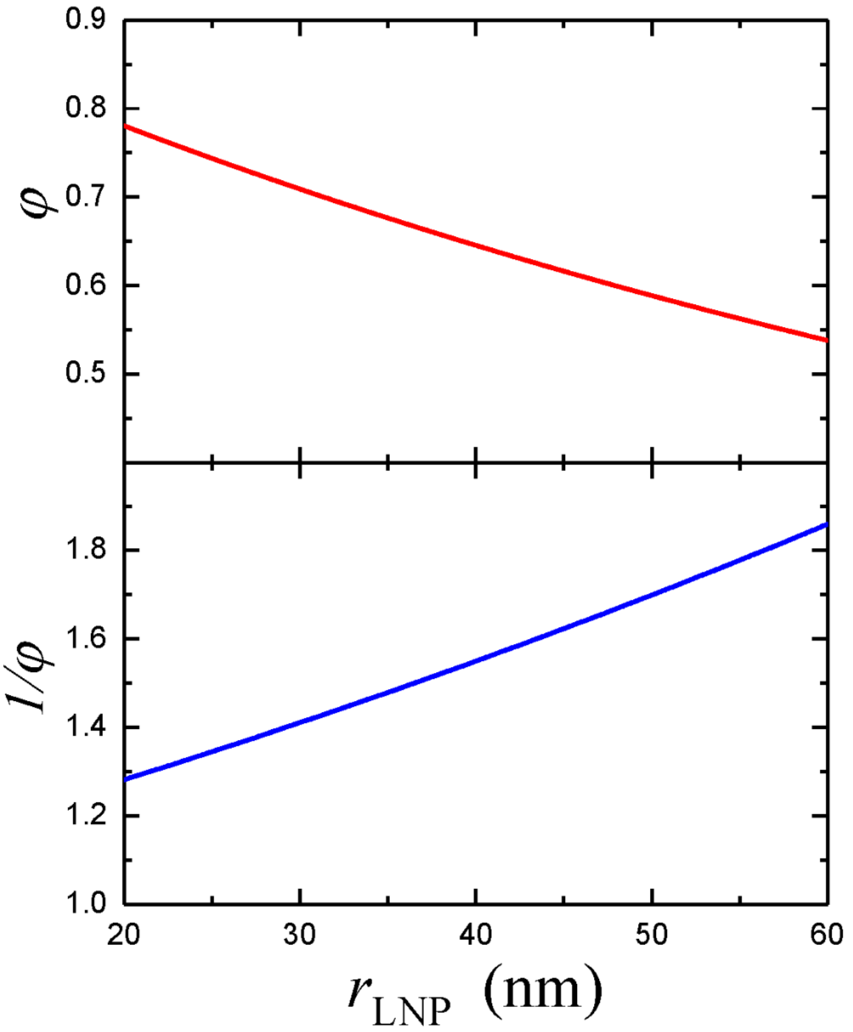

Figure S8. Factors φ and $1/\varphi$ as a function of the LNP radius. The former factor characterizes the decrease of the LNPs' TIRF intensity due to the drop of the evanescence field. The latter factor characterizes the increase of the TIRF intensity upon the LNP collapse. The calculations have been performed by using Equation 2 with δ = 100 nm and l = 5 nm.

---

9. Interaction between LNPs in solution

To understand whether LNPs can aggregate in biofluids containing primarily water, it is instructive to estimate the interaction between them. It can be done in the framework of the conventional DLVO theory representing this interaction as a sum of the van der Waals (vdW), hydration, and double-layer electrostatic parts, U_{vdW}, U_h, and U_{dl}.[6] The former two forces operate on the length scale of ≃ 1 nm, while the range of the latter forces depends on the NP size and is

appreciably larger. In general, all these forces should be considered. Bearing in mind the physiological conditions, one can, however, notice that the double-layer interaction between identical LNPs is usually rather weak and repulsive (provided the associated charges are of the same sign) and does not result in aggregation. In addition, the parameters available to calculate this interaction are not accurate. For these reasons, we exclude U_{dl} from our analysis and represent the interaction between two LNPs as

$$U = U_{\mathrm{vdW}} + U_{\mathrm{h}}. \tag{3}$$

To calculate U_{vdW}, we use the conventional additive Hamaker approximation (see ref. 7 and references therein),

$$U_{\mathrm{vdW}} = -A_{\mathrm{LNP-LNP}}\phi(R_1, R_2, d)/6, \tag{4}$$

where $A_{\mathrm{LNP\text{-}LNP}}$ is the Hamaker constant, d is the minimal NP-NP distance, and

$$\phi(R_1, R_2, d) \equiv \frac{2R_1R_2}{2(R_1+R_2)d+d^2} + \frac{2R_1R_2}{4R_1R_2+2(R_1+R_2)d+d^2} + \ln\left[\frac{2(R_1+R_2)d+d^2}{4R_1R_2+2(R_1+R_2)d+d^2}\right]. \tag{5}$$

The corresponding hydration energy is given by [7]

$$U_{\mathrm{h}} = \frac{2\pi B R_1 R_2}{\alpha(R_1+R_2)} \exp(-\alpha d), \tag{6}$$

where α and B are the parameters determined via the energy of the interaction (per unit area) of the flat interfaces, $U_{\mathrm{h}} = B\exp(-\alpha d)$.

To calculate the vdW interaction, we need the Hamaker constant for the lipid phase. According to measurements and calculations,[8] a reasonable value of this constant is $A_{\mathrm{LNP-LNP}} = 0.5\times10^{-20}$ (or $\simeq 1.3\ k_{\mathrm{B}}T$). To describe the hydration energy, we use $B = 0.03$ J/m^2 and $\alpha = 3.8$ nm^{-1}.[7]

The LNP-LNP interaction, calculated with these parameters as a function of d for the LNP sizes of interest or, more specifically, for the average radius, 40 nm, and maximum radius, 60 nm, is relatively weak (Figure S9) and cannot result in aggregation of LNPs in solution.

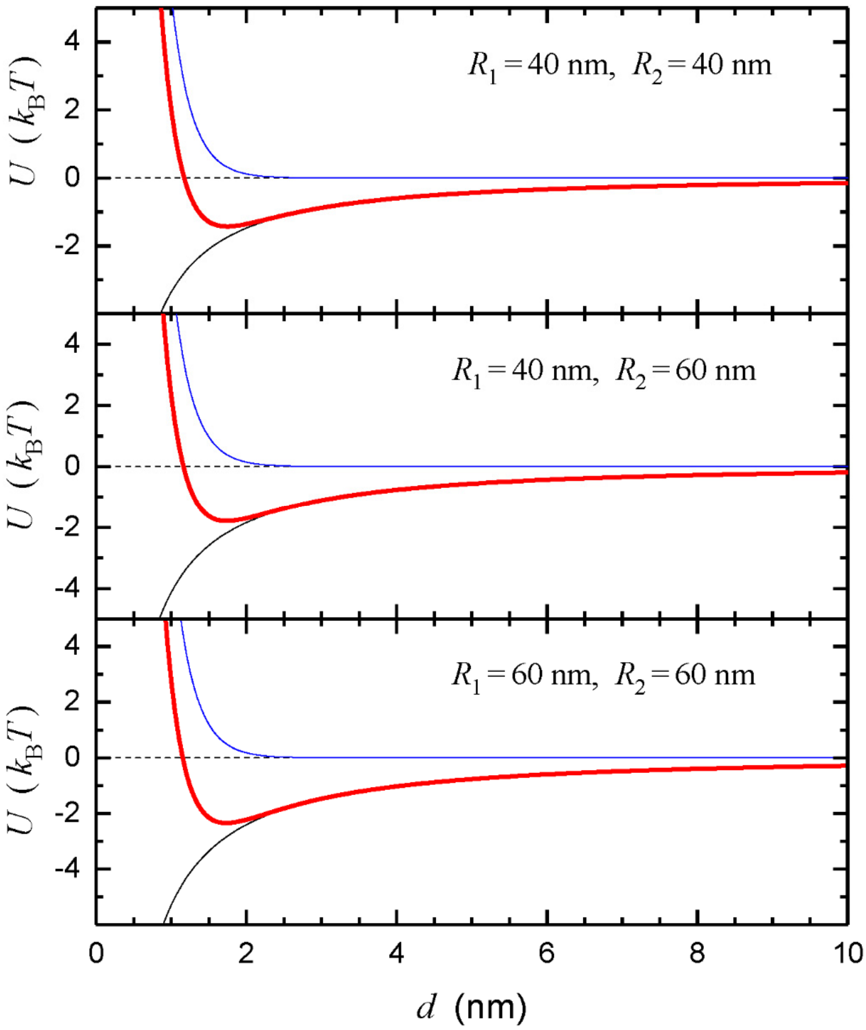

Figure S9. Energy of the interaction between LNPs as a function of the minimal distance between them. The van der Waals and hydration interaction are shown by black and blue thin solid lines, respectively. The whole interaction is represented by the red thick solid line. The LNP sizes are indicated in the panels.

This conclusion holds even if $A_{\text{LNP-LNP}}$ is increased by a factor of 2 (this factor characterizes the accuracy of the used value of $A_{\text{LNP-LNP}}$). Hence, for aggregation to occur as the pH drops would require structural changes that either result in heterogeneous distributions of surface charge on the LNPs and/or a heterogeneous LNP sample with LNPs with both positive and negative average charge.

10. Specifics of measurements under flow conditions

Our experiments were performed under flow conditions. In adsorption experiments with biological molecules (e.g. proteins) or nanoparticles (e.g. vesicles or LNP as in our case), such conditions are preferable because the attachment of such species is often globally controlled by diffusion, and the analysis of the corresponding diffusion-limited adsorption kinetics under the flow conditions is simpler than that under the stagnant (no macroscopic flow) conditions.

Regarding the flow conditions, one can wonder whether the flow influences the rate of kinetic processes occurring locally near the interfaces. This effect is usually axiomatically assumed to be negligible. To validate this assumption in our cases, we can notice that the scale of the solution velocity near the interface or, more specifically, around an attached LNP is given by

$$u \cong vR/L, \qquad (7)$$

where v is the maximum flow rate, L is the scale of the channel size in the direction perpendicular to the flow, and R is the LNP radius. According to the Stokes model,[9] the scale of the viscosity-related force acting on a LNP is

$$F \cong 6\pi\eta Ru, \qquad (8)$$

where η is the coefficient of dynamic viscosity. Substituting (5) into (6) yields

$$F \cong 6\pi\eta R^2 v/L. \qquad (9)$$

Using Eq. (9) with η = 1×10^{-3} Ns/m^2, v = 1.6×10^{-3} m/s, L = 400 μm, and R = 40 nm (see Materials and Methods for volumetric flow rate and channel dimensions), we obtain F < 1 fN. For comparison, one can notice that the forces corresponding to attachment or reconfiguration of LNPs are usually much larger than around 10 $k_B T/a$ = 20 pN ($a \cong 2$ nm is the corresponding length scale). The latter force is much larger than that estimated above by using Eq. (9), and accordingly the effect of the solution flow on the processes under consideration is negligible.

11. References

(1) Jayaraman, M.; Ansell, S. M.; Mui, B. L.; Tam, Y. K.; Chen, J.; Du, X.; Butler, D.; Eltepu, L.; Matsuda, S.; Narayanannair, J. K.; Rajeev, K. G.; Hafez, I. M.; Akinc, A.; Maier, M. A.; Tracy, M. A.; Cullis, P. R.; Madden, T. D.; Manoharan, M.; Hope, M. J. Maximizing the Potency of SiRNA Lipid Nanoparticles for Hepatic Gene Silencing In Vivo. *Angewandte Chemie International Edition* **2012**, *51* (34), 8529–8533.

(2) Havel, R. J.; Eder, H. A.; Bragdon, J. H. The Distribution and Chemical Composition of Ultracentrifugally Separated Lipoproteins in Human Serum. *J Clin. Invest* **1955**, *34* (9), 1345–1353.

(3) Arteta, M. Y.; Kjellman, T.; Bartesaghi, S.; Wallin, S.; Wu, X.; Kvist, A. J.; Dabkowska, A.; Székely, N.; Radulescu, A.; Bergenholtz, J.; Lindfors, L. Successful Reprogramming of Cellular Protein Production through MRNA Delivered by Functionalized Lipid Nanoparticles. *Proc. Natl. Acad. Sci. U.S.A.* **2018**, *115* (15), E3351–E3360.

(4) Gallud, A.; Munson, M. J.; Liu, K.; Idström, A.; Barriga, H. M. G.; Tabaei, S. R.; Aliakbarinodehi, N.; Ojansivu, M.; Lubart, Q.; Doutch, J. J.; Holme, M. N.; Evenäs, L.; Lindfors, L.; Stevens, M. M.; Collén, A.; Sabirsh, A.; Höök, F.; Esbjörner, E. K. Time Evolution of PEG-Shedding and Serum Protein Coronation Determines the Cell Uptake Kinetics and Delivery of Lipid Nanoparticle Formulated mRNA; *bioRxiv* **2021**; DOI: 10.1101/2021.08.20.457104

(5) Rupert, D. L. M.; Shelke, G. V.; Emilsson, G.; Claudio, V.; Block, S.; Lässer, C.; Dahlin, A.; Lötvall, J. O.; Bally, M.; Zhdanov, V. P.; Höök, F. Dual-Wavelength Surface Plasmon Resonance for Determining the Size and Concentration of Sub-Populations of Extracellular Vesicles. *Anal. Chem.* **2016**, *88* (20), 9980–9988.

(6) Israelachvili, J. N. *Intermolecular and Surface Forces*; 3rd Edition, Academic Press, Burlington, 2011.

(7) Zhdanov, V. P. Nanoparticles without and with Protein Corona: Van Der Waals and Hydration Interaction. *J. Biol. Phys.* **2019**, *45* (3), 307–316.

(8) Walz, J. Y.; Ruckenstein, E. Comparison of the van Der Waals and Undulation Interactions between Uncharged Lipid Bilayers. *The Journal of Physical Chemistry B* **1999**, *103* (35), 7461–7468.

(9) Landau, L. D. and Lifshitz, E. M. *Fluid Mechanics; Pergamon Press*: Oxford, 1987.
